# Rectangular Coordination Polymer Nanoplates: Large-Scale, Rapid Synthesis and Their Application as a Fluorescent Sensing Platform for DNA Detection

**DOI:** 10.1371/journal.pone.0030426

**Published:** 2012-01-18

**Authors:** Yingwei Zhang, Yonglan Luo, Jingqi Tian, Abdullah M. Asiri, Abdulrahman O. Al-Youbi, Xuping Sun

**Affiliations:** 1 State Key Lab of Electroanalytical Chemistry, Changchun Institute of Applied Chemistry, Changchun, Jilin, People's Republic of China; 2 Chinese Academy of Sciences, Graduate School of the Chinese Academy of Sciences, Beijing, People's Republic of China; 3 Chemistry Department, Faculty of Science, King Abdulaziz University, Jeddah, Saudi Arabia; 4 Center of Excellence for Advanced Materials Research, King Abdulaziz University, Jeddah, Saudi Arabia; University of Helsinki, Finland

## Abstract

In this paper, we report on the large-scale, rapid synthesis of uniform rectangular coordination polymer nanoplates (RCPNs) assembled from Cu(II) and 4,4′-bipyridine for the first time. We further demonstrate that such RCPNs can be used as a very effective fluorescent sensing platform for multiple DNA detection with a detection limit as low as 30 pM and a high selectivity down to single-base mismatch. The DNA detection is accomplished by the following two steps: (1) RCPN binds dye-labeled single-stranded DNA (ssDNA) probe, which brings dye and RCPN into close proximity, leading to fluorescence quenching; (2) Specific hybridization of the probe with its target generates a double-stranded DNA (dsDNA) which detaches from RCPN, leading to fluorescence recovery. It suggests that this sensing system can well discriminate complementary and mismatched DNA sequences. The exact mechanism of fluorescence quenching involved is elucidated experimentally and its use in a human blood serum system is also demonstrated successfully.

## Introduction

Simple, fast, cost-effective, and sensitive detection of specific DNA sequences is crucial to molecular diagnostics for pathogen detection and biomedical research. The past years have witnessed the growing importance in developing specific methods for DNA detection, which has various applications in gene expression profiling, clinical disease diagnostics and treatment, fast detection of biological warfare agents, and forensic applications etc [1]. Detecting genetic mutations at the molecular level opens up the possibility of performing reliable disease diagnostics in clinical practice even before any symptom of a disease appears. Polymerase chain reaction (PCR) as a technique for DNA amplification and sequencing has found extensive application in modern biological and medical sciences; however, it has the disadvantages of high cost, risk of contamination, and false-negative results [2], [3]. Gene chip is a widely used high-throughput DNA detection technique, but it requires highly precise and expensive instrumentation for fluorescent signal readout and needs sophisticated numerical algorithms to interpret the data [4]. Thus, new DNA detection methods need to be developed. Many efforts have recently been made to develop homogeneous fluorescence assays based on FRET (fluorescence resonance energy transfer) or quenching mechanism for DNA sequence detection [5]. It is shown that nanostructures can be used as a quencher in this assay with the advantage of eliminating the selection issue of fluorophore-quencher because they can quench dyes of different emission frequencies [5], [6]. Until now, we and other researchers have successfully demonstrated that versatile structures can serve as an effective quencher for fluorescence-enhanced DNA detection, including gold nanoparticles [7]–[11], single-walled carbon nanotubes (SWCNTs) [12], carbon nanoparticles [13], nano-C_60_ [14], graphene oxide (GO) [15], [16], poly(*p*-phenylenediamine) nanobelts (PNs) [17], poly(*m*-phenylenediamine) (PMPD) nanorods [18], Ag@poly(m-phenylenediamine) core−shell nanoparticles [19], polyaniline nanofibres [20], poly(*o*-phenylenediamine) colloids [21], supramolecular microparticles [22], etc. However, all the above systems have their inherent drawbacks which limit their practical use. For example, the SWCNT or GO system suffers from the high cost that both SWCNT and graphite powder used for producing GO are usually purchased from some manufacturers and suppliers, and on the other hand, an organic solvent like N,N-dimethylformamide (DMF) is used to disperse SWCNT by a period of several hours sonication or the GO preparation by the Hummer's method is time-consuming and labor-intensive [12], [23]. Our PN system has the disadvantage that the nanobelts are tens of micrometers in length and tend to sink in the aqueous solution due to the gravity, causing the problem of stability in detection [17]. An additional limitation is that its discrimination ability toward complementary and single-base mismatched target sequences is very poor.

Coordination polymers (CPs) are a class of organic−inorganic hybrid materials, in which metal ions are linked together by organic bridging ligands, and have been developed extremely rapidly due to their versatile properties provoked by combining the merits of two sources and may find applications in many fields [24]–[29]. Only until recently, however, have CPs been used for DNA detection where coordination polymer colloids and nanobelts were used as a quencher [30]–[32], but these systems still suffer from more or less severe drawbacks, including (1) the H_2_PtCl_6_ precursor is expensive and monodispersed structures can't be prepared on a large scale [30]–[32]; (2) a period of several hours is required to prepare the colloids [30]; (3) the dye fluorescence can't be completely quenched by these quenchers, leading to strong background fluorescence [30]–[32]; (4) the gradual reduction of Ag(I) by 4,4′-bipyridine with elapsed time leads to Ag nanoparticle-decorated nanobelts [31], [33]. Accordingly, the development of new fluorescent sensing platform overcoming all the above-mentioned shortcomings is highly desirable.

In this paper, for the first time, we report on the large-scale, rapid, and economic synthesis of uniform rectangular coordination polymer nanoplates (RCPNs) assembled from Cu(II) and 4,4′-bipyridine, carried out by directly mixing aqueous CuCl_2_ solution and ethanol solution of 4,4′-bipyridine at room temperature. We further demonstrate that such RCPNs can be used as a very effective fluorescent sensing platform for multiple DNA detection with a detection limit as low as 30 pM and a high selectivity down to single-base mismatch. The DNA detection is accomplished by the following two steps: (1) RCPN binds dye-labeled single-stranded DNA (ssDNA) probe, which brings dye and RCPN into close proximity, leading to fluorescence quenching. (2) Specific hybridization of the probe with its target generates a double-stranded DNA (dsDNA) which detaches from RCPN, leading to fluorescence recovery. It suggests that this sensing system can well discriminate complementary and mismatched DNA sequences. The exact mechanism of fluorescence quenching involved is elucidated experimentally and its use human blood serum system is also demonstrated successfully.

## Results and Discussion

**Figure 1:**
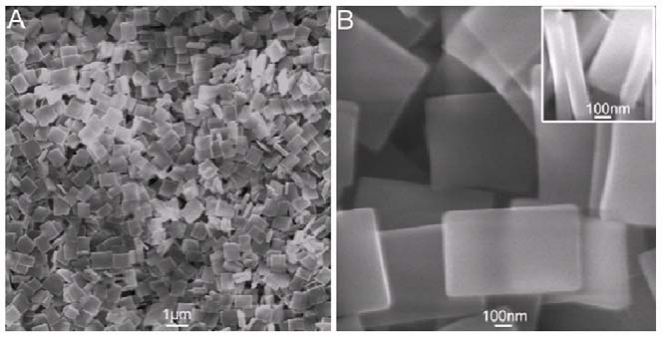
Instrumental analysis of the precipitate thus formed. (A) Low and (B) high magnification SEM images of the precipitates thus formed. Inset shows that the nanoplate is about 100 nm in thickness.

The mixing of aqueous CuCl_2_ solution and ethanol solution of 4,4′-bipyridine results in the immediate formation of a large amount of blue precipitates (see **Materials and Methods** for preparation details). Figure 1A shows the low magnification SEM image of the precipitates thus formed, indicating that the products consist exclusively of a large quantity of rectangular coordination nanoplates. The high magnification image further reveals that these plates are nanoplates with typical dimensions of 800 nm in length, 500 nm in width, and 100 nm in thickness (inset), as shown in Figure 1B. Figure S1 gives the histogram of sizes of these nanoplates with standard deviation. The chemical composition of these nanoplates was determined by energy-dispersive spectrum (EDS) shown in Figure S2. The peaks of C, N, Cu, and Cl elements are observed, indicating that these nanoplates are products of 4,4′-bipyridine and CuCl_2_. It is well-documented that nitrogen-contained ligand can coordinate with Cu(II) [34]. We can suggest that the formation of such rectangular coordination polymer nanoplates in our present study is attributed to coordination-induced assembly from Cu(II) and 4,4′-bipyridine. A possible formation mechanism of the nanoplates is briefly presented as following. When Cu(II) and 4,4′-bipyridine are mixed together, the two nitrogen atoms on the para positions of one 4,4′-bipyridine molecule can coordinate to two different Cu(II) cations leading to 4,4′-bipyridine-bridged structure, and the Cu species contained in as-formed structure can further capture other 4,4′-bipyridine molecules by coordination interactions along different directions. This coordination-induced assembly process can proceed repeatedly until the depletion of reactants in the solution, resulting in the formation of large coordination polymers, finally. The formation of such RCPNs is complete within seconds and thus it is impossible for us to trace the time-dependent growth process. We can only speculate the possible formation mechanism involved and the detailed formation mechanism is far beyond our understanding. It is of importance to note that the yield of RCPNs is estimated to be about 98% based on the weight difference between the reactants and products.

**Figure 2:**
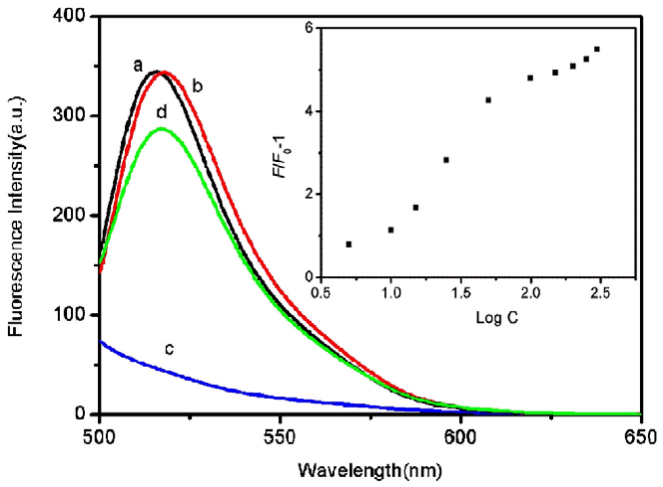
Performance of target DNA detection. Fluorescence emission spectra of P_HIV_ (50 nM) at different conditions: (a) P_HIV_; (b) P_HIV_ + 300 nM T_1_; (c) P_HIV_ + RCPNs; (d) P_HIV_ + RCPNs + 300 nM T_1_. Inset: fluorescence intensity ratio of P_HIV_-RCPN complex with *F*/*F*_0_–1 (*F*_0_ and *F* are the fluorescence intensity without and with the presence of T_1_, respectively) plotted against the logarithm of the concentration of T_1_ (nM). Excitation was at 480 nm, and the emission was monitored at 518 nm. All measurements were done in Tris-HCl buffer in the presence of 15 mM Mg^2+^ (pH: 7.4).

To test the feasibility of using RCPNs as a novel effective fluorescent sensing platform for nucleic acid detection, an oligonucleotide sequence associated with human immunodeficiency virus (HIV) was chosen as a model system. Figure 2 shows the fluorescence emission spectra of P_HIV_ at different conditions. The fluorescence spectrum of P_HIV_, the FAM-labeled probe, in the absence of RCPNs, exhibits strong fluorescence emission due to the presence of the fluorescein-based dye (curve a). However, in the presence of RCPNs, we failed to see the fluorescence emission peak of the FAM dye (curve c), indicating that RCPNs can adsorb ssDNA and quench the fluorescent dye very effectively. On the other hand, the P_HIV_-RCPN complex shows significant fluorescence enhancement upon its incubation with complementary target T_1_ over a period of 1 h, leading to about 83% fluorescence recovery (curve d). It should be noted that the fluorescence intensity of the free P_HIV_ was, however, scarcely influenced by the addition of T_1_ in the absence of RCPNs (curve b). Figure 2 inset illustrates the fluorescence intensity changes (*F*/*F*_0_–1) of P_HIV_-RCPN complex upon addition of different concentrations of T_1_, where *F*_0_ and *F* are FAM fluorescence intensities at 518 nm in the absence and the presence of T_1_, respectively. In the DNA concentration range of 5–300 nM, a dramatic increase of FAM fluorescence intensity was observed, suggesting that the RCPN/DNA assembly approach is effective in probing biomolecular interactions.

**Figure 3:**
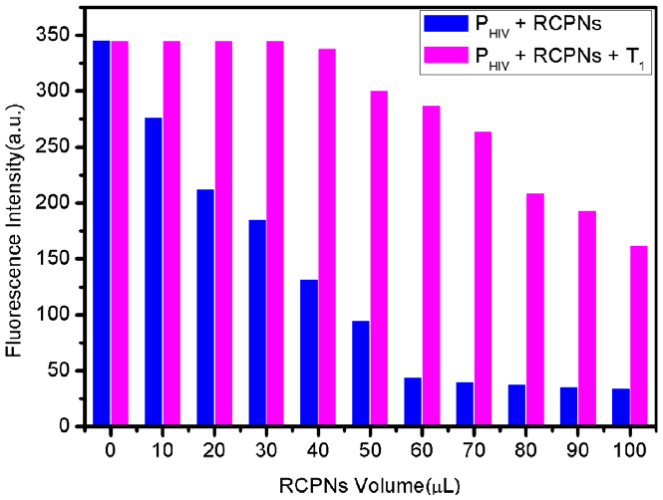
Investigation of the influence of the amount of RCPNs on the system. Fluorescence intensity histograms of P_HIV_ + RCPNs and P_HIV_ + RCPNs + T_1_ with the use of 0, 10, 20, 30, 40, 50, 60, 70, 80, 90, 100 µL of RCPNs in this system ([P_HIV_] = 50 nM; [T_1_] = 300 nM). Excitation was at 480 nm, and the emission was monitored at 518 nm. All measurements were done in Tris-HCl buffer in the presence of 15 mM Mg^2+^ (pH: 7.4).

It was found that the amount of RCPNs involved in this system has profound effect on the efficiency of the fluorescence quenching and the subsequent recovery. The increase of RCPNs in volume from 0 to 100-µL leads to increased quenching efficiency but a decrease in recovery efficiency (Figure 3). Thus, an optimal volume of 60-µL was chosen in our present study if not specified. It is also important to note that optimal signal-to-noise ratio and hence lower detection limit can be obtained by decreasing the amount of RCPN and P_HIV_ used._._For instance, a detection limit as low as 30 pM was achieved when the experiment was carried out using 0.6-µL RCPNs and 500 pM P_HIV_ in the system (Figure S3). It is of importance to note that such RCPNs can be well-dispersed in water by shaking and kept stable during our measurements.

Such RCPN is a π-rich structure and thus there should be strong π-π stacking interactions between ssDNA bases and RCPN [35]. Indeed, we have found that fluorescence quenching was suppressed by introducing N,N-dimethylformamide (DMF) to change the solvent polarity in assay solution (Figure S4), which can be attributed to that this π-π interaction is weaken by DMF molecules leading to decreased adsorption of ssDNA on RCPNs [19]. The zeta potential of the nanoplate was measured to be about 24.1 mV in pure water, meaning the nanoplate has a positively charged surface due to the presence of Cu(II) cations in the plate. However, the electrostatic attractive interactions between RCPN and negatively charged backbone of ssDNA is greatly weakened due to the presence of a large amount of salt in buffer [19]. In contrast, RCPN should have weak or no binding with dsDNA due to the absence of unpaired DNA bases and the rigid conformation of dsDNA. Scheme S1A presents a schematic to illustrate the fluorescence-enhanced nucleic acid detection using RCPN as a sensing platform. The DNA detection is accomplished by the following two steps: In the first step, the adsorption of fluorescent FAM-labeled ssDNA onto the nanoplate via π-π stacking leads to fluorescence quenching due to their close proximity. In the second step, the specific hybridization of the dye-labeled ssDNA with its target leads to fluorescence recovery because the hybridization will disturb the interaction between the dye-labeled ssDNA and nanoplate, producing a dsDNA which detaches from RCPN. The release of the dsDNA from RCPNs is evidenced by the following experimental fact that no obvious fluorescence change was observed after the removal of the RCPNs from the solution by centrifugation, as shown in Figure S5. The absorption spectrum of RCPNs dispersed in Tris-HCl buffer (pH 7.4) shown in Figure S6 exhibits an absorption peak at 238 nm, suggesting that there is no spectra overlap and thus no FRET occurs between RCPN and the fluorescent dye FAM. We can attribute the observed fluorescence quenching in our present study to photoinduced electron transfer (PET) from nitrogen atom in RCPN to excited fluorophore due to their close proximity [36]. Scheme S1B presents a schematic to illustrate the fluorescence quenching mechanism involved. When the fluorophore is excited, an electron from the highest occupied molecular orbital (HOMO) is promoted to the lowest unoccupied molecular orbital (LUMO), leaving an electronic vacancy in the fluorophore HOMO, which is filled by transfer of an electron from the higher energy HOMO of the nitrogen atom in RCPN serving as a donor. The overall effect of PET process is that the excited state lifetime is shortened and little fluorescence occurs. Note that although the pyridine binds to Cu(II) via coordination of its N atom to Cu center, given that 4,4′-bipyridine has two N atoms at opposite positions, it is expected that there are still some N atoms available on the RCPN surface for protonation. Upon protonation of the donor, its redox potential is raised and its HOMO becomes lower in energy than that of the fluorophore. Consequently, electron transfer from donor to fluorophore is hindered and the fluorescence quenching is thus suppressed. Although fluorescence intensity of dye-labeled probe is sensitive to pH value, it was observed that fluorescence quenching is suppressed with the increase of protonation degree of donor by decreasing the pH value of the system (Figure S7).

**Figure 4:**
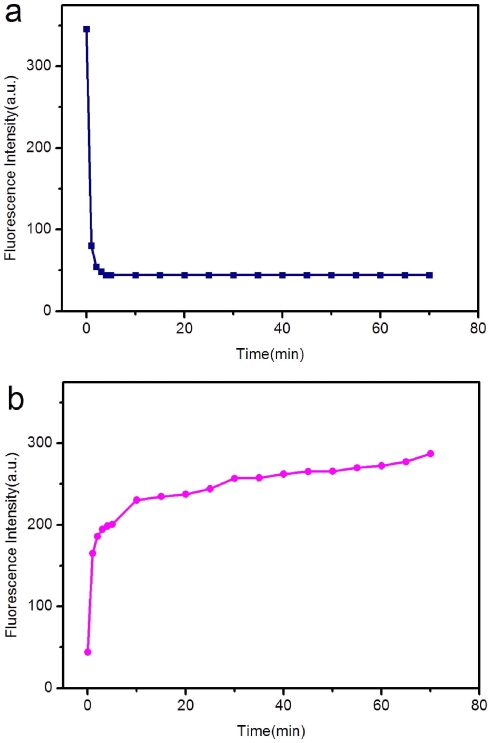
Kinetic behaviour study of fluorescence quenching and recovery at different temperatures. (a) Fluorescence quenching of P_HIV_ (50 nM) by RCPNs and (b) fluorescence recovery of P_HIV_-RCPN by T_1_ (300 nM) as a function of incubation time. Excitation was at 480 nm, and the emission was monitored at 518 nm. All measurements were done in Tris-HCl buffer in the presence of 15 mM Mg^2+^ (pH: 7.4).

We also studied the kinetic behaviors of P_HIV_ and RCPNs, as well as of the P_HIV_-RCPN complex with T_1_ by collecting the time-dependent fluorescence emission spectra (Figure 4). Figure 4A shows the fluorescence quenching of P_HIV_ in the presence of RCPNs as a function of incubation time. In the absence of the target, the curve exhibits a rapid reduction in the first 1 min and reaches equilibrium within the following 4 min, indicating that ssDNA adsorption on RCPN is much faster than on SWCNT but similar to on GO [6], [12], [14], [15]. Figure 4B shows the fluorescence recovery of P_HIV_-RCPN by T_1_ as a function of time. In the presence of the target T_1_, the curve shows a fast increase in the first 1 min, followed by a slow fluorescence enhancement. The best fluorescence response was obtained after about 70 min of incubation time. It suggests that the kinetics of the hybridization of the probe adsorbed on RCPN to its target and the subsequent release of the dsDNA thus formed from RCPN resembles that on GO but faster than on SWCNT [6], [12], [14], [15]. In addition, the fluorescence quenching data are also analyzed by the Stern-Volmer equation: *F*_0_/*F* = 1 + *K*_SV_[*Q*] [37], where *F*_0_ and *F* are the steady-state fluorescence intensities in the absence and presence of quencher (RCPNs), respectively. *K*_SV_ is the Stern-Volmer quenching constant, and [*Q*] the concentration of quencher. The constant *K*sv thus provides a direct measure of the quenching sensitivity. Hence, we have performed a quencher concentration-dependent quenching study to calculate *K*_SV_ of RCPNs, and compared with that of PNs, CNTs and GO, as shown in Figure S8. Table S1 shows the Stern-Volmer quenching constant *K*_SV_ of FAM fluorescence by different quenchers. These results indicate that the RCPNs have relatively higher quenching sensitivity.

**Figure 5:**
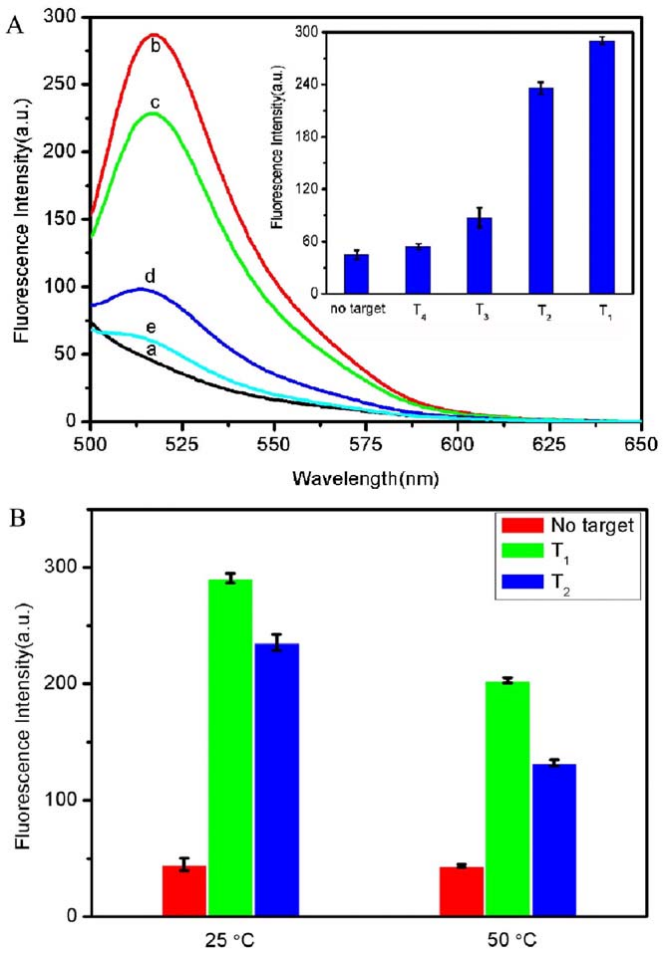
Evaluation of discrimination ability at different temperatures. (A) Fluorescence emission spectra of P_HIV_ (50 nM) at different conditions: (a) P_HIV_-RCPN complex; (b) P_HIV_-RCPN complex + 300 nM T_1_; (c) P_HIV_-RCPN complex + 300 nM T_2_; (d) P_HIV_-RCPN complex + 300 nM T_3_; (e) P_HIV_-RCPN complex + 300 nM T_4_. Inset: fluorescence intensity histograms with error bar. (B) Fluorescence signal enhancement of P_HIV_-RCPN complex upon incubation with T_1_ and T_2_ at 25 and 50°C, respectively. Excitation was at 480 nm, and the emission was monitored at 518 nm. All measurements were done in Tris-HCl buffer in the presence of 15 mM Mg^2+^ (pH: 7.4). The error bar represents the standard deviation of three measurements.

The discrimination ability of the present sensing platform toward complementary and mismatched target sequences was further investigated. Figure 5A shows the fluorescence responses of PHIV-RCPN complex toward complementary target T_1_, single-base mismatched target T_2_, two-base mismatched target T_3_, and non-complementary target T_4_. It is observed that the *F*/*F*_0_ value (*F*_0_ and *F* are the fluorescence intensities without and with the presence of target, respectively) obtained upon addition of 300 nM of T_2_ and T_3_ is about 79% and 33% of the value obtained upon addition of 300 nM of T_1_ into the P_HIV_-RCPN complex, respectively. In contrast, only very small fluorescence change was observed for the P_HIV_-RCPN upon addition of 300 nM T_4_, indicating that the fluorescence enhancement in our present system is indeed due to the base pairing between probe and its target other than competitive binding. Compared to the complementary target, the mismatched target should have lower hybridization ability toward the ssDNA probe. As a result, a decreased hybridization and thus fluorescence recovery efficiency is observed. Figure 5A inset shows the corresponding fluorescence intensity histograms with error bar. We also carried out hybridization experiments at an elevated temperature of 50°C. It should be noted that FAM-ssDNA only exhibits slight fluorescence decrease at elevated temperature. The observed decrease of FAM fluorescence intensity at elevated temperature in our present study can be attributed to that hybridization stringency conditions do not favor duplex formation between short single strands [17], [38], leading to decreased hybridization and thus fluorescence recovery efficiency. It is found that the *F*/*F*_0_ value obtained upon addition of T_2_ is about 56% of the value obtained upon addition of T_1_ into P_HIV_-RCPN complex. Figure 5B compares the fluorescence signal enhancement of P_HIV_-RCPN complex upon incubation with T_1_ and T_2_ at 25 and 50°C, respectively. All the above observations indicate that the present sensing system can well discriminate complementary and mismatched DNA sequences with good reproducibility and its discrimination ability increases with increased hybridization temperature which makes the hybridization harder for probe and mismatched target.

**Figure 6:**
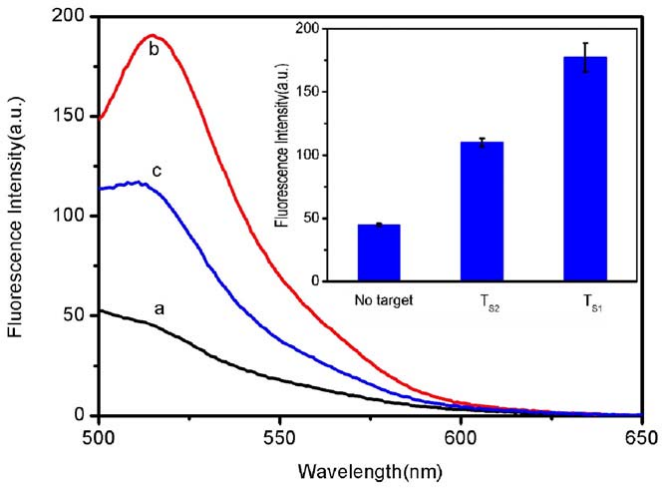
Evaluation of discrimination ability using shorter probe. Fluorescence emission spectra of P_S_ (50 nM) at different conditions: (a) P_S_-RCPN complex; (b) P_S_-RCPN complex + 300 nM T_S1_; (c) P_S_-RCPN complex + 300 nM T_S2_. Inset: fluorescence intensity histograms with error bar. Excitation was at 480 nm, and the emission was monitored at 518 nm. All measurements were done in Tris-HCl buffer in the presence of 15 mM Mg^2+^ (pH: 7.4). The error bar represents the standard deviation of three measurements.

It was found that the use of shorter oligonucleotide can further improve the ability of this sensing system to distinguish mismatch. Figure 6 shows the fluorescence responses of FAM-labeled, 9-nucleotide ssDNA probe P_S_ (50 nM) toward complementary target T_S1_ and single-base mismatched target T_S2_ in the presence of RCPNs at room temperature. The *F*/*F*_0_ value obtained upon addition of 300 nM of T_S2_ is about 59% of the value obtained upon addition of 300 nM of T_S1_ into P_S_-RCPN complex.

**Figure 7:**
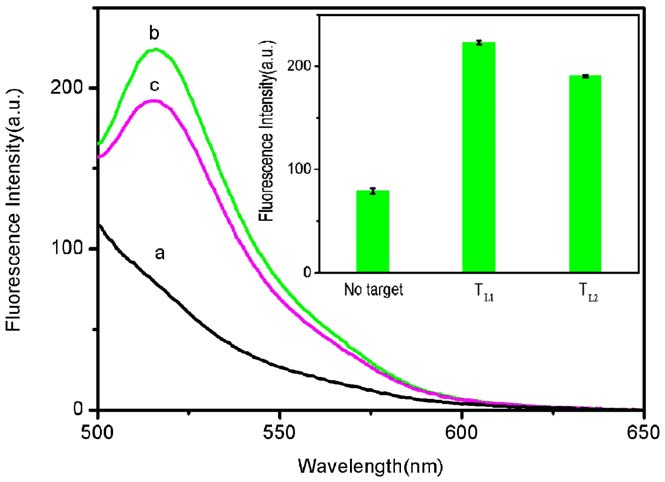
Evaluation of discrimination ability using longer target. Fluorescence emission spectra of P_HIV_ (50 nM) in the presence of 40-µL RCPNs at different conditions: (a) P_HIV_-RCPN complex; (b) P_HIV_-RCPN complex + 300 nM T_L1_; (c) P_HIV_-RCPN complex + 300 nM T_L2_. Inset: fluorescence intensity histograms with error bar. Excitation was at 480 nm, and the emission was monitored at 518 nm. All measurements were done in Tris-HCl buffer in the presence of 15 mM Mg^2+^ (pH: 7.4). The error bar represents the standard deviation of three measurements.

The discrimination ability of this sensing platform was also evaluated by detecting specific sequences on much longer DNA targets than probes. For this purpose, two long DNA strands were chosen as targets: T_L1_, the middle part of which is complementary sequence to P_HIV_; T_L2_, the middle part of which is single-base mismatched sequence to P_HIV_. Figure 7 shows the fluorescence responses of P_HIV_ toward T_L1_ and T_L2_ in the presence of RCPNs at room temperature. The addition of T_L1_ to P_HIV_-RCPN complex leads to about 65% fluorescence recovery which is much lower than 83% observed when T_1_ was used as the target. It can be attributed to that P_HIV_-T_L1_ is a complex with a duplex segment in the middle and two single strands on both ends and thus there are unpaired DNA bases for binding to RCPN. The *F*/*F*_0_ value obtained upon addition of 300 nM of T_L2_ is about 84% of the value obtained upon addition of 300 nM of T_L1_ into P_HIV_-RCPN complex, indicating that this sensing platform is still capable of distinguishing complementary and single-base mismatched target sequence in a large DNA strand with a short oligonucleotide probe.

**Figure 8:**
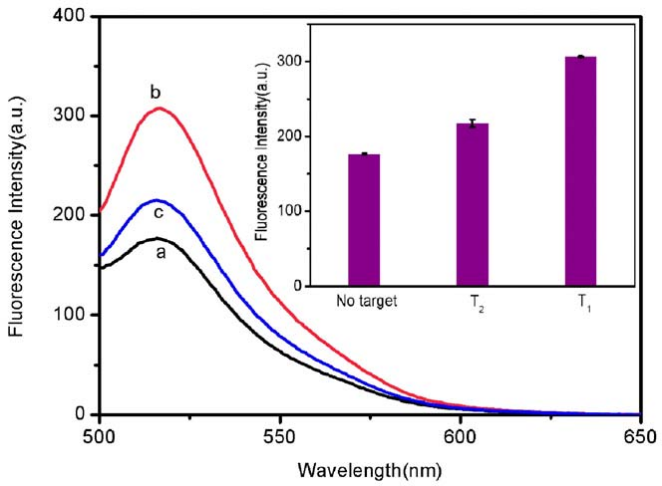
Performance of single-base mismatch discrimination in the presence of blood serum. Fluorescence emission spectra of P_HIV_ (50 nM) in 40-µL blood serum and 360-µL Tris-HCl buffer containing 15 mM Mg^2+^ (pH: 7.4) at different conditions: (a) P_HIV_ + RCPNs; (b) P_HIV_ + RCPNs + 300 nM T_1_; (c) P_HIV_ + RCPNs + 300 nM T_2_. Inset: fluorescence intensity histograms with error bar. Excitation was at 480 nm, and the emission was monitored at 518 nm. The error bar represents the standard deviation of three measurements.

From the above results, it can be clearly seen that this RCPN-based sensing platform has excellent sensitivity and selectivity in pure buffer systems. In further experiments, the potential application of this sensing platform for real sample analysis was challenged with human blood serum samples. Figure 8 shows the fluorescence emission spectra of P_HIV_ in the presence of 10% blood serum (volume ratio) in Tris-HCl buffer at different conditions. The *F*/*F*_0_ value obtained upon addition of 300 nM of T_2_ is about 70% of the value obtained upon addition of 300 nM of T_1_ into P_HIV_-RCPN complex, indicating that this system is still capable of distinguishing complementary and mismatched sequences with good reproducibility in the presence of blood serum. These observations show that our measurements were not seriously affected by blood serum components and hence this sensing system is likely to be capable of practically useful mismatch detection upon further development.

**Figure 9:**
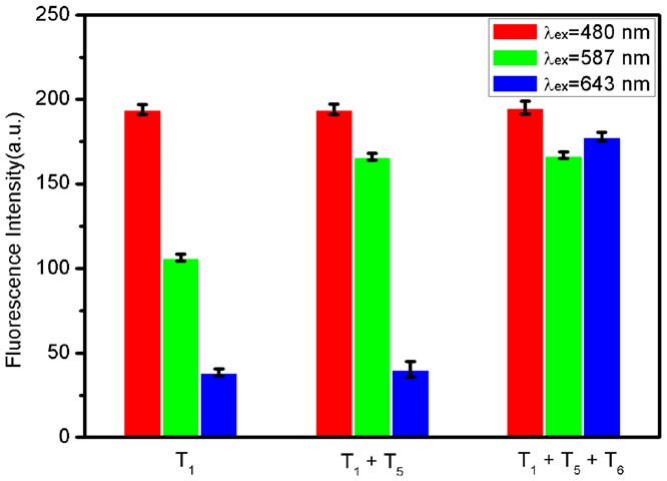
Multiplex DNA detection. Fluorescence intensity histograms of the probe mixture toward different target combinations in the presence of RCPNs under excitation/emission wavelengths of 480/518, 587/615, and 643/660 nm/nm. All measurements were done in Tris-HCl buffer in the presence of 15 mM Mg^2+^ (pH: 7.4). The error bar represents the standard deviation of three measurements.

Finally, the feasibility of using the platform described herein to detect multiple DNA targets simultaneously was explored. To this end, we chose FAM-labeled P_HIV_ and another two probes P_HBV_ and P_K167_ labeled with ROX and Cy5 (cyanine 5), respectively, as model systems. Because these three dyes are individually excited at 480, 587, and 643 nm to emit at 518, 615, and 660 nm, respectively, significant dye-to-dye energy transfer is avoided. In the presence of RCPNs, the fluorescence of all dyes in the probe mixture was heavily quenched, suggesting that RCPN can effectively quench dyes of different emission frequencies. Figure 9 shows the fluorescence intensity histograms of the probe mixture toward different target combinations in the presence of RCPNs under excitation/emission wavelengths of 480/518, 587/615, and 643/660 nm/nm. It is clearly seen that the addition of T_1_ gives only one strong emission peak at 518 nm when excited at 480 nm. However, the target combination of T_1_+T_5_ gives two strong emission peaks at 518 and 615 nm when excited at 480 and 587 nm, respectively. Three strong emission peaks are observed for the T_1_+T_5_+T_6_ target combination at 518, 615, and 660 nm when excited at 480, 587, and 643 nm, respectively. Based on all the above observations, it can be concluded that this sensing platform can be used for multiple nucleic acid detection.

In summary, uniform RCPNs have been rapidly prepared on a large scale at room temperature and further used as a very effective fluorescent sensing platform for multiple DNA detection with high sensitivity and selectivity. The mechanism of fluorescence quenching is discussed and the application of this sensing platform in human blood serum system is also demonstrated successfully. This RCPN-based assay is homogenous, “mix and read” and requires no wash or complex sample preparation steps. Our present observations are significant for the following three reasons: (1) it provides us a facile method for the rapid, economic synthesis of RCPNs for DNA detection; (2) such RCPNs are obviously easier to produce and thus the RCPN-based DNA sensing platform is practically more important than those based on other structures [5]–[12]; (3) this RCPN-based assay holds great promise for practical application in clinical sample analysis.

## Materials and Methods

All chemically synthesized oligonucleotides were purchased from Shanghai Sangon Biotechnology Co. Ltd. (Shanghai, China). DNA concentration was estimated by measuring the absorbance at 260 nm. All the other chemicals were purchased from Aladin Ltd. (Shanghai, China) and used as received without further purification. The water used throughout all experiments was purified through a Millipore system. Human blood serum was obtained form Institute of Virology and AIDS Research, First Affiliated Hospital, Jilin University, Changchun, Jilin, People's Republic of China.

Oligonucleotide sequences used are listed below (mismatch underlined):

- [1] P_HIV_ (FAM dye-labeled ssDNA):

5′-FAM-AGT CAG TGT GGA AAA TCT CTA GC-3′

- [2] T_1_ (complementary target to P_HIV_):

5′-GCT AGA GAT TTT CCA CAC TGA CT-3′

- [3] T_2_ (single-base mismatched target to P_HIV_):

5′-GCT AGA GAT TGT CCA CAC TGA CT-3′

- [4] T_3_ (two-base mismatched target to P_HIV_):

5′-GCT AGA GAT TGT ACA CAC TGA CT-3′

- [5] T_5_ (non-complementary target to P_HIV_):

5′-TTT TTT TTT TTT TTT TTT TTT TT-3′

- [6] P_s_ (FAM dye-labeled shorter ssDNA):

5′-TGG AAA ATC-3′

- [7] T_s1_ (complementary target to P_s_):

5′-GAT TTT CCA-3′

- [8] T_s2_ (single-base mismatched target to P_s_):

5′-GAT TGT CCA-3′

- [9] P_HBV_ (ROX dye-labeled ssDNA):

5′-ROX-TAC CAC ATC ATC CAT ATA ACT GA-3′

- [10] T_5_ (complementary target to P_HBV_):

5′-TCA GTT ATA TGG ATG ATG TGG TA-3′

- [11] P_K167_ (Cy5 dye-labeled ssDNA):

5′-Cy5-TCT GCA CAC CTC TTG ACA CTC CG-3′

- [12] T_6_ (complementary target to P_K167_):

5′-CGG AGT GTC AAG AGG TGT GCA GA-3′

- [13] T_L1_ (The middle part of a long strand as a target complementary to P_HIV_):

5′-TTT TTT TTT TTT TTT TTT TTT TGC TAG AGA TTT TCC ACA CTG ACT TTT TTT TTT TTT TTT TTT TTT T-3′

- [14] T_L2_ (The middle part of a long strand as a single-base mismatched target to P_HIV_):

5′-TTT TTT TTT TTT TTT TTT TTT TGC TAG AGA TTG TCC ACA CTG ACT TTT TTT TTT TTT TTT TTT TTT T-3′

The RCPNs were prepared as follows: In brief, 1 mL of 0.1 M 4,4′-bipyridine ethanol solution was added into 9 mL of 5.6 mM CuCl_2_ aqueous solution at room temperature under stirring. After that, a large amount of blue precipitates occurred immediately. The resulting precipitates were washed with water by centrifugation twice first, and then re-dispersed in 5 mL of water for characterization and further use.

Scanning electron microscopy (SEM) measurements were made on a XL30 ESEM FEG scanning electron microscope at an accelerating voltage of 20 kV. Fluorescent emission spectra were recorded on a RF-5301PC spectrofluorometer (Shimadzu, Japan). Zeta potential measurements were performed on a Nano-ZS Zetasizer ZEN3600 (Malvern Instruments Ltd., U.K.). For characterization by scanning electron microscopy (SEM), 20 µL of the suspension was placed on an indium tin oxide (ITO) glass slide and air-dried at room temperature. Scanning electron microscopy (SEM) measurements were made on a XL30 ESEM FEG scanning electron microscope at an accelerating voltage of 20 kV. An energy-dispersive X-ray spectroscopic detecting unit was used to collect the energy-dispersed spectrum (EDS) for elemental analysis. The volume of each sample for fluorescence measurement is 400 µL in 20 mM Tris-HCl buffer containing 100 mM NaCl, 5 mM KCl, and 15 mM MgCl_2_ (pH: 7.4). All the experiments were carried out at room temperature (about 25°C) if not specified.

## Supporting Information

Figure S1 **Sizes distribution determination.** The histogram of sizes of the coordination polymer nanoplates.
(TIF)

Figure S2 **Chemical composition analysis.** Energy-dispersive spectrum of the coordination polymer nanoplates.
(TIF)

Figure S3 **Determination of detection limit.** (a) Fluorescence quenching of P_HIV_ (500 pM) by RCPNs and (b) fluorescence recovery of P_HIV_-RCPN by T_1_ (30 pM). Inset: fluorescence intensity histograms with error bar. Excitation was at 480 nm, and the emission was monitored at 518 nm. All measurements were done in Tris-HCl buffer in the presence of 15 mM Mg^2+^ (pH: 7.4).
(TIF)

Figure S4 **Investigation of the influence of the solvent polarity on the system.** Fluorescence intensity histograms of P_HIV_ (50 nM) in the presence of RCPNs at different solvent conditions: (a) 300 µL Tris-HCl buffer and (b) 150 µL Tris-HCl buffer + 150 µL DMF.
(TIF)

Figure S5 **Confirmation of** t**he release of the dsDNA from RCPNs.** Fluorescence emission spectra of (a) P_HIV_-RCPN complex + T_1_ and (b) the supernatant of (a) after removing RCPNs by centrifugation. ([P_HIV_] = 50 nM; [T_1_] = 300 nM; *λ*_ex_ = 480 nm). All measurements were done in Tris-HCl buffer in the presence of 15 mM Mg^2+^ (pH: 7.4).
(TIF)

Figure S6 **UV-Vis absorption of RCPNs.** Absorption spectrum of RCPNs dispersed in Tris-HCl buffer in the presence of 15 mM Mg^2+^ (pH 7.4).
(TIF)

Figure S7 **Investigation of the influence of pH value on the fluorescence quenching.** The histograms of fluorescence intensity changes (1–*F*/*F*_0_) of FAM-labeled ssDNA at different pH values, where *F*_0_ and *F* are fluorescence intensities at 518 nm in the absence and presence of RCPNs, respectively.
(TIF)

Figure S8 **Stern–Volmer quenching constant (K_SV_) determination of different quenchers.** Stern-Volmer plot for quenching of the FAM fluorescence by different quenchers at room temperature: (a) graphene oxide (GO); (b) multi-walled carbon nanotubes (MWNT); (c) poly(*p*-phenylenediamine) nanobelts (PNs); (d) RCPNs.
(TIF)

Scheme S1 **Illustration of the sensing process and fluorescence quenching mechanism.** A schematic (not to scale) to illustrate (A) the fluorescence-enhanced nucleic acid detection using RCPN as a sensing platform and (B) the PET-based fluorescence quenching mechanism.
(TIF)

Table S1 **Stern-Volmer quenching constant** ***K*** **_SV_ of FAM fluorescence by different quenchers at room temperature.**
(TIF)
