# Supplementary figures and images for: Rectangular Coordination Polymer Nanoplates: Large-Scale, Rapid Synthesis and Their Application as a Fluorescent Sensing Platform for DNA Detection
Source: PLoS One. 2012 Jan 18;7(1):e30426. doi: 10.1371/journal.pone.0030426 (PMC3261194; doi:10.1371/journal.pone.0030426)

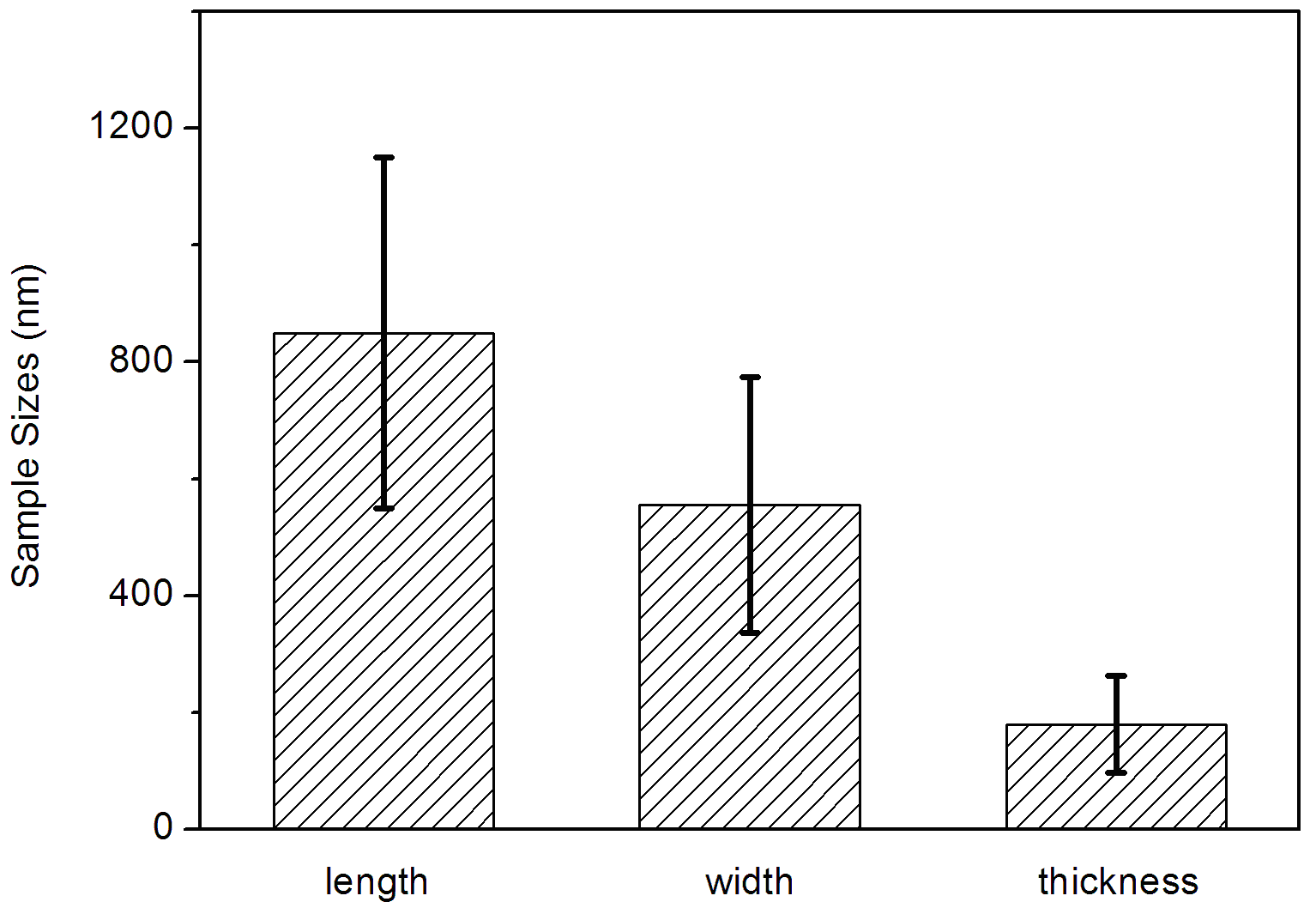

Supplement: Figure S1 — Sizes distribution determination. The histogram of sizes of the coordination polymer nanoplates. (TIF) [file pone.0030426.s001.tif]

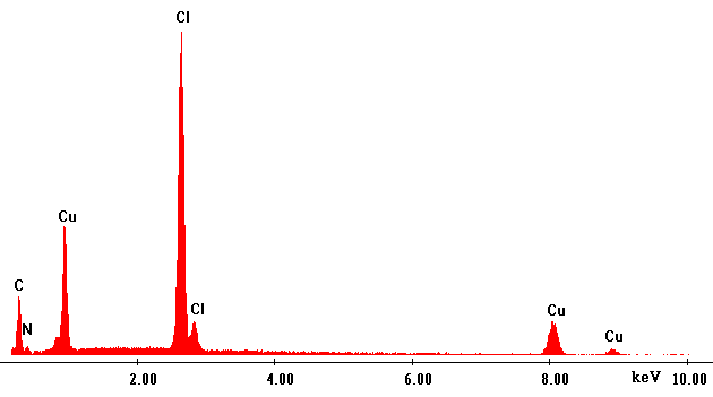

Supplement: Figure S2 — Chemical composition analysis. Energy-dispersive spectrum of the coordination polymer nanoplates. (TIF) [file pone.0030426.s002.tif]

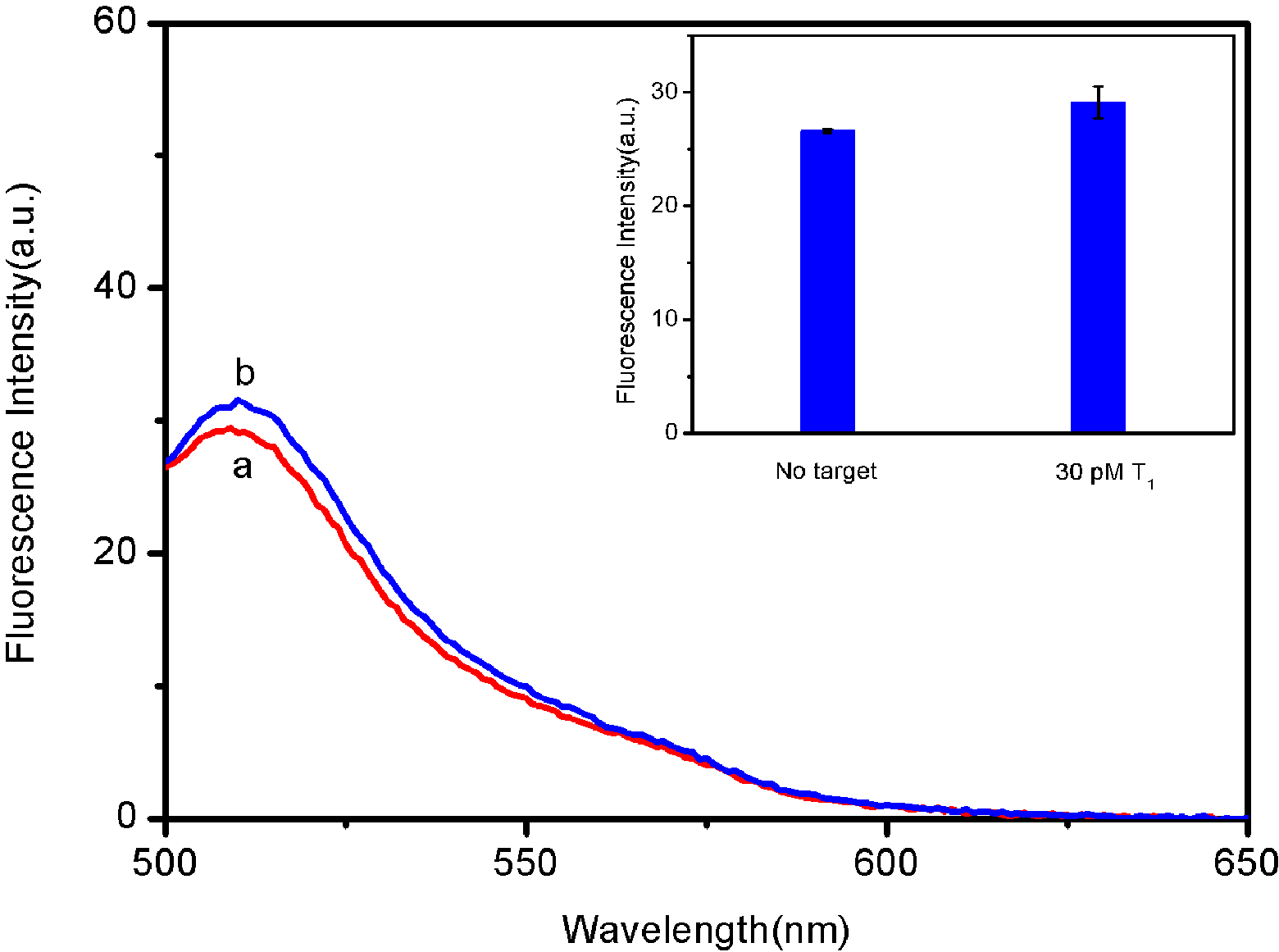

Supplement: Figure S3 — Determination of detection limit. (a) Fluorescence quenching of PHIV (500 pM) by RCPNs and (b) fluorescence recovery of PHIV-RCPN by T1 (30 pM). Inset: fluorescence intensity histograms with error bar. Excitation was at 480 nm, and the emission was monitored at 518 nm. All measurements were done in Tris-HCl buffer in the presence of 15 mM Mg2+ (pH: 7.4). (TIF) [file pone.0030426.s003.tif]

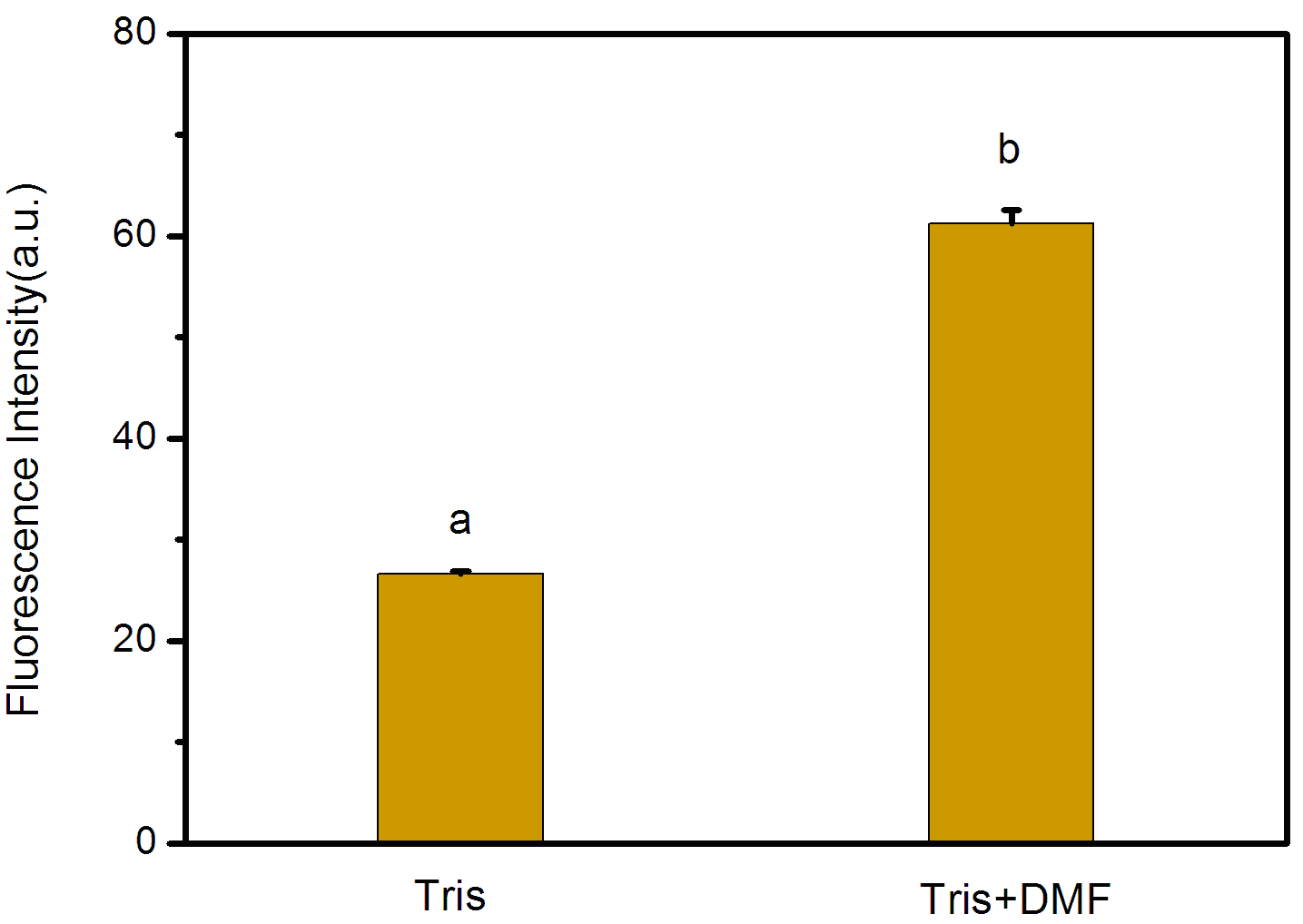

Supplement: Figure S4 — Investigation of the influence of the solvent polarity on the system. Fluorescence intensity histograms of PHIV (50 nM) in the presence of RCPNs at different solvent conditions: (a) 300 µL Tris-HCl buffer and (b) 150 µL Tris-HCl buffer + 150 µL DMF. (TIF) [file pone.0030426.s004.tif]

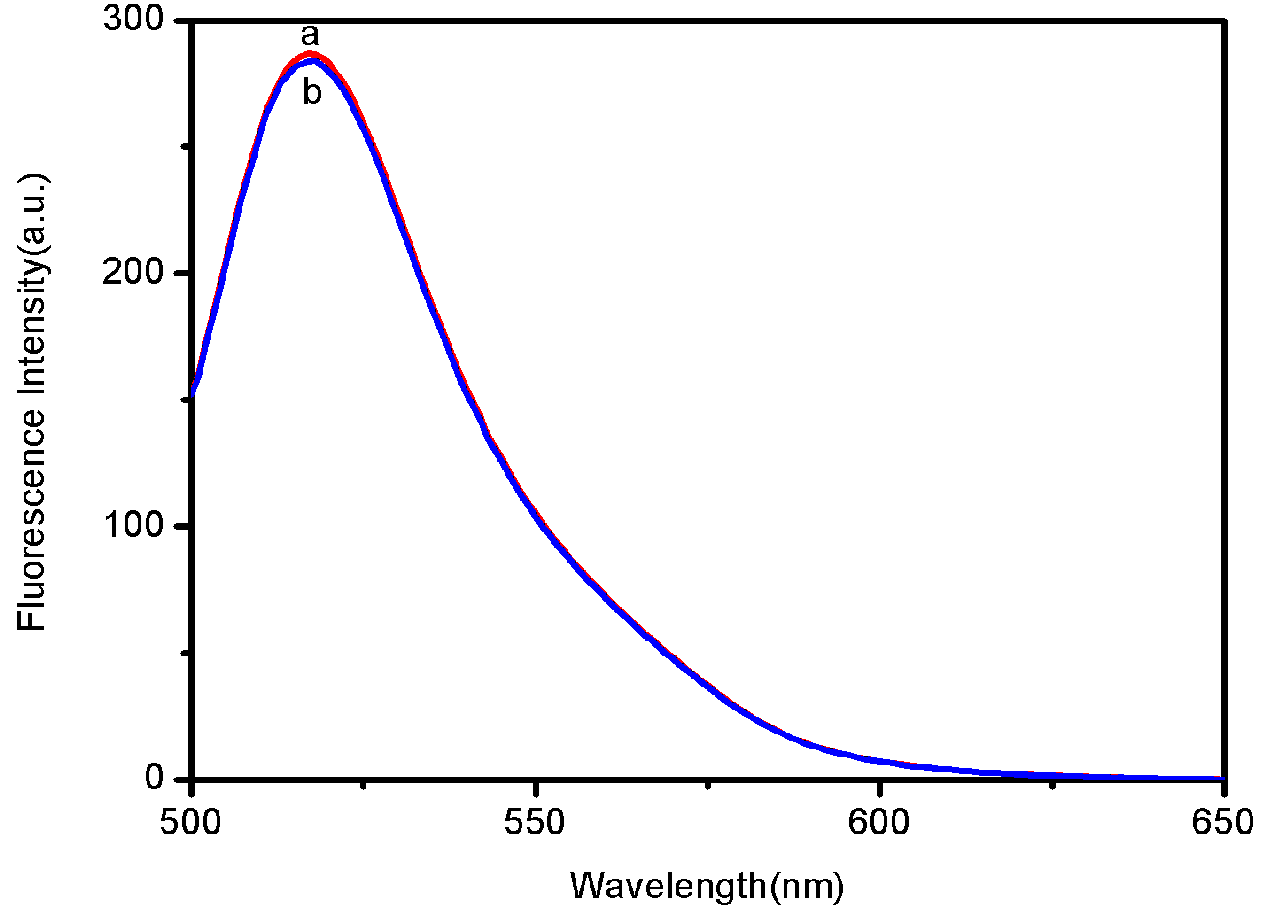

Supplement: Figure S5 — Confirmation of the release of the dsDNA from RCPNs. Fluorescence emission spectra of (a) PHIV-RCPN complex + T1 and (b) the supernatant of (a) after removing RCPNs by centrifugation. ([PHIV] = 50 nM; [T1] = 300 nM; λ ex = 480 nm). All measurements were done in Tris-HCl buffer in the presence of 15 mM Mg2+ (pH: 7.4). (TIF) [file pone.0030426.s005.tif]

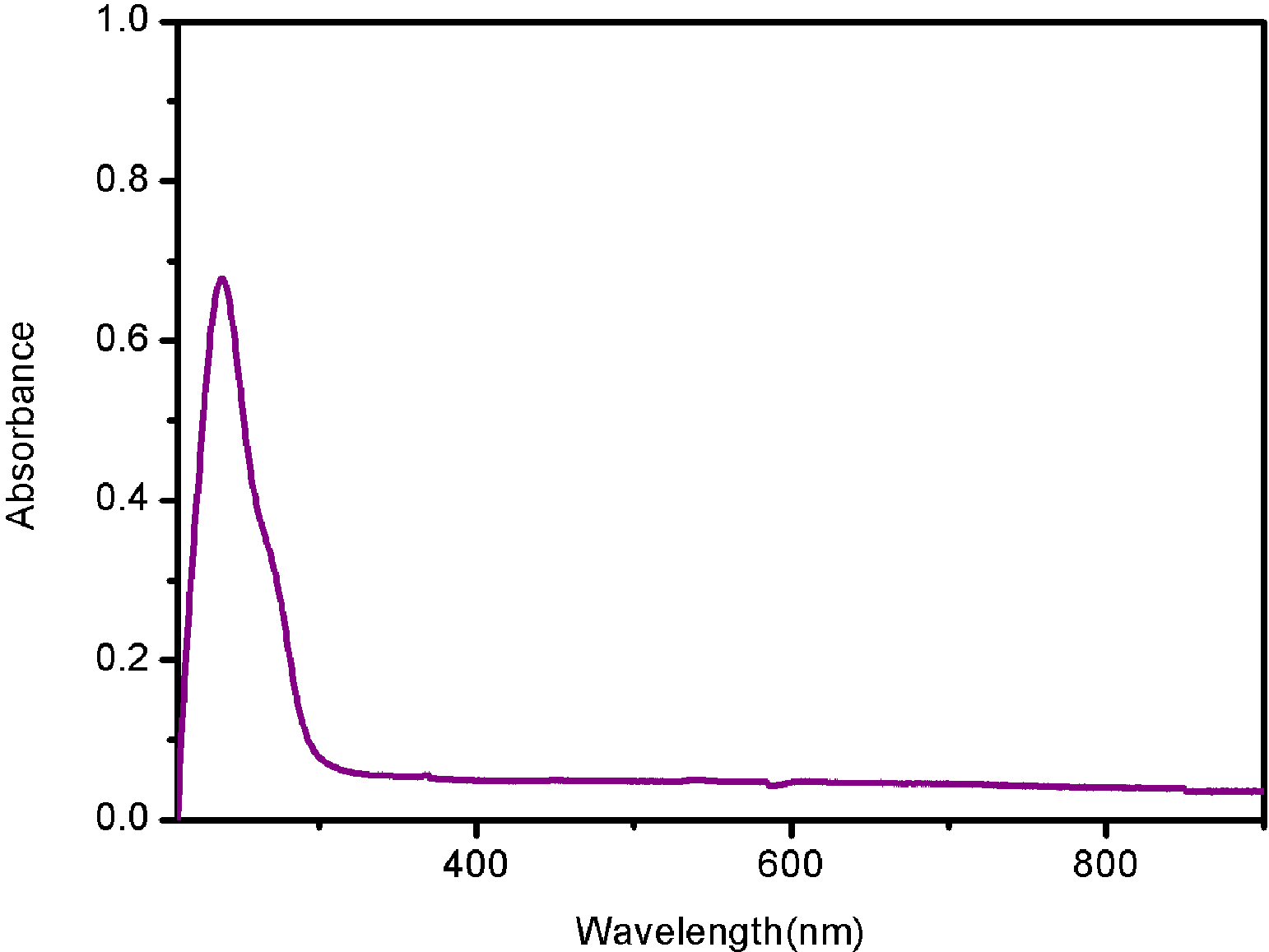

Supplement: Figure S6 — UV-Vis absorption of RCPNs. Absorption spectrum of RCPNs dispersed in Tris-HCl buffer in the presence of 15 mM Mg2+ (pH 7.4). (TIF) [file pone.0030426.s006.tif]

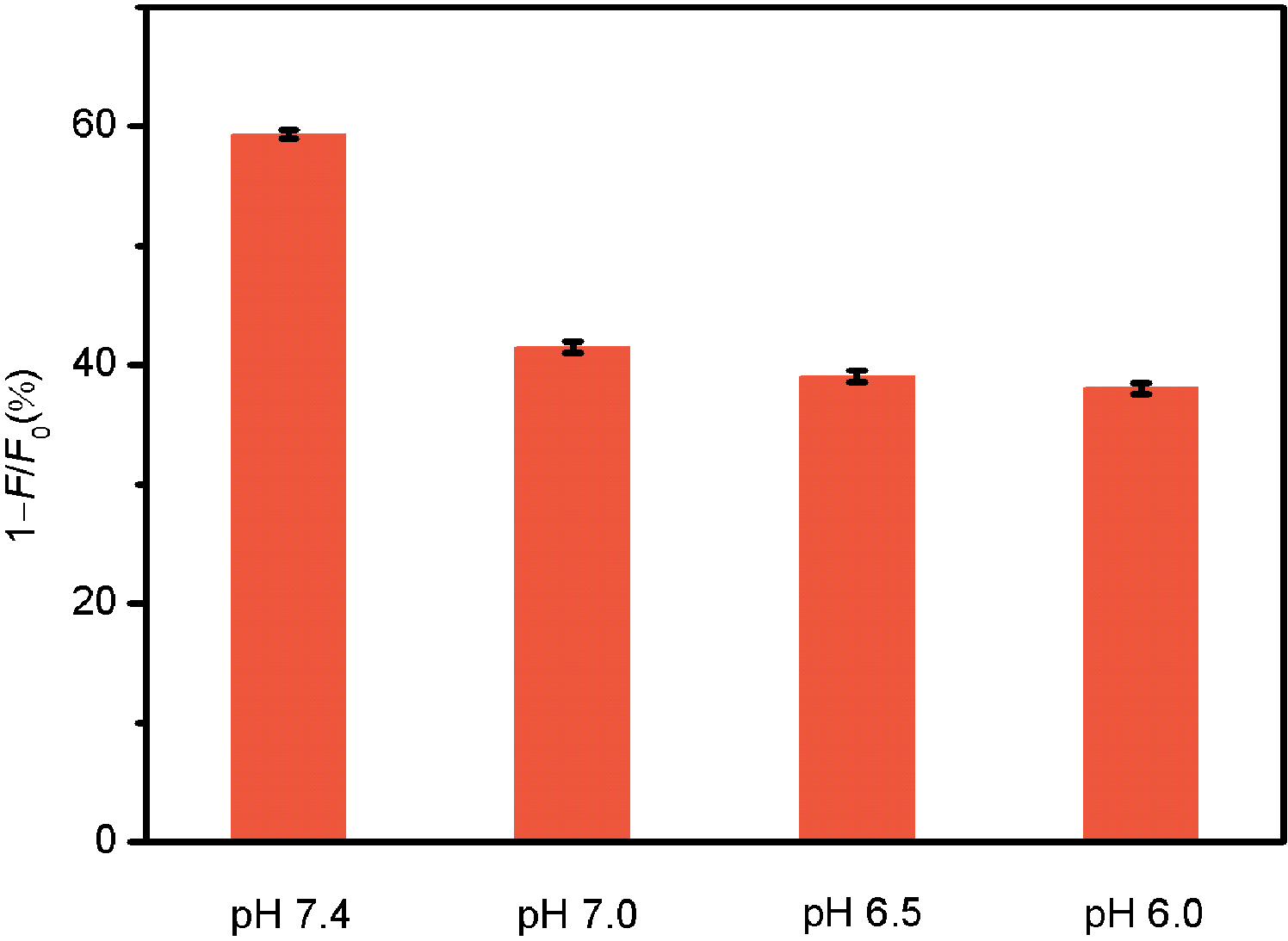

Supplement: Figure S7 — Investigation of the influence of pH value on the fluorescence quenching. The histograms of fluorescence intensity changes (1–F/F 0) of FAM-labeled ssDNA at different pH values, where F 0 and F are fluorescence intensities at 518 nm in the absence and presence of RCPNs, respectively. (TIF) [file pone.0030426.s007.tif]

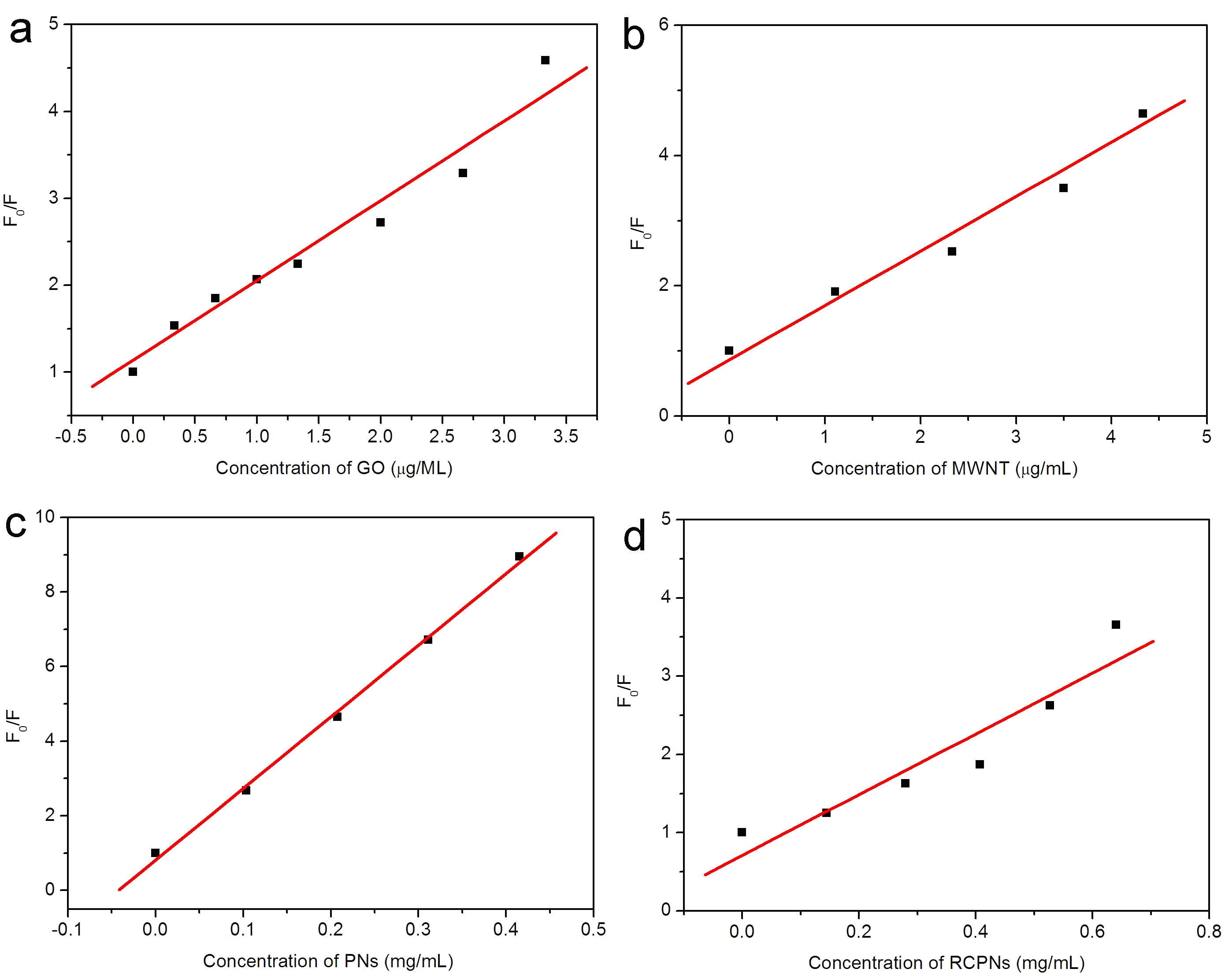

Supplement: Figure S8 — Stern–Volmer quenching constant (KSV) determination of different quenchers. Stern-Volmer plot for quenching of the FAM fluorescence by different quenchers at room temperature: (a) graphene oxide (GO); (b) multi-walled carbon nanotubes (MWNT); (c) poly(p-phenylenediamine) nanobelts (PNs); (d) RCPNs. (TIF) [file pone.0030426.s008.tif]

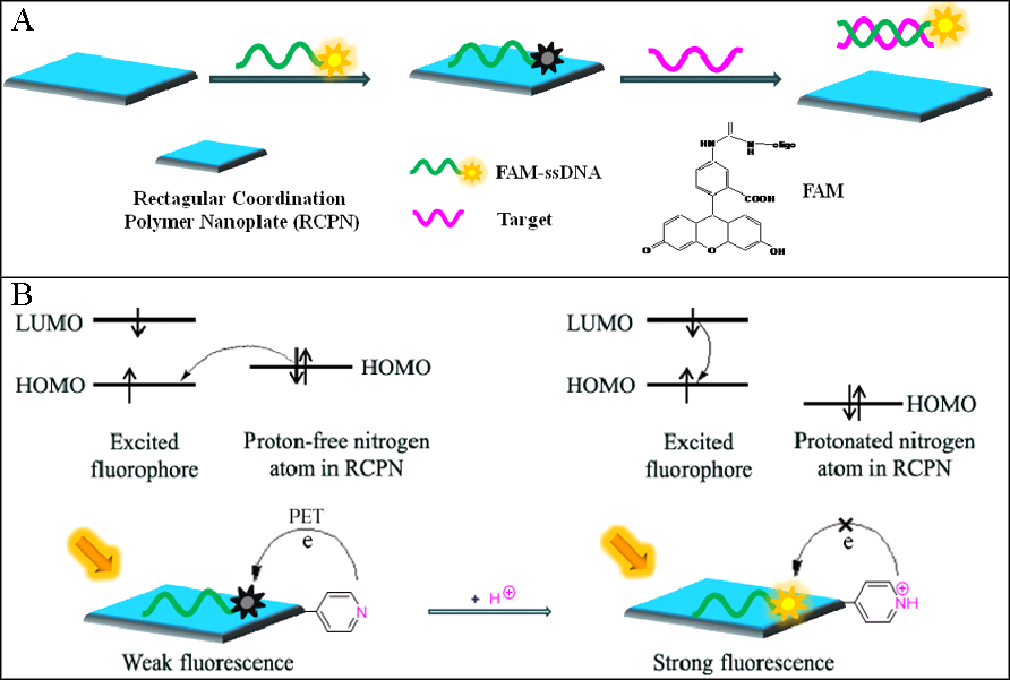

Supplement: Scheme S1 — Illustration of the sensing process and fluorescence quenching mechanism. A schematic (not to scale) to illustrate (A) the fluorescence-enhanced nucleic acid detection using RCPN as a sensing platform and (B) the PET-based fluorescence quenching mechanism. (TIF) [file pone.0030426.s009.tif]

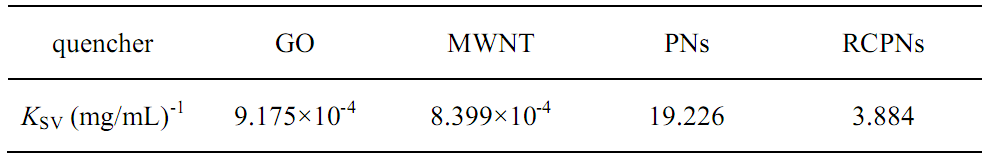

Supplement: Table S1 — Stern-Volmer quenching constant K SV of FAM fluorescence by different quenchers at room temperature. (TIF) [file pone.0030426.s010.tif]
